# SVT quest: The adventure diagnosing narrow QRS tachycardia

**DOI:** 10.1002/joa3.13112

**Published:** 2024-07-11

**Authors:** Koichi Nagashima, Gregory F. Michaud, Reginald T. Ho, Yasuo Okumura

**Affiliations:** ^1^ Division of Cardiology, Department of Medicine Nihon University School of Medicine Tokyo Japan; ^2^ Division of Cardiovascular Medicine Massachusetts General Hospital Boston Massachusetts USA; ^3^ Division of Cardiology, Department of Medicine Thomas Jefferson University Hospital Philadelphia Pennsylvania USA

**Keywords:** atrial tachycardia, atrioventricular nodal reentrant tachycardia, nodoventricular pathway, orthodromic reciprocating tachycardia, supraventricular tachycardia

## Abstract

In the field of cardiac electrophysiology, there is a universal desire: the discovery of a flawless diagnostic maneuver for supraventricular tachycardias (SVTs). This is not merely a wish but a shared odyssey. To improve diagnostic accuracy and achieve sufficient sensitivity and specificity, numerous diagnostic maneuvers have been proposed. However, each has its limitations and prompts a search for new diagnostic techniques. This continuous cycle of discovery and refinement, which we titled “SVT Quest” is reviewed in chronological sequence. This adventure in diagnosing narrow QRS tachycardia unfolds in 3 steps: Step 1 involves differentiating atrial tachycardia from other SVTs based on the observations such as V‐A‐V or V‐A‐A‐V response, ΔAA interval, VA linking, the last entrainment sequence, and response to the atrial extrastimulus. Step 2 focuses on differentiating orthodromic reciprocating tachycardia from atrioventricular nodal reentrant tachycardia based on the observations such as tachycardia reset upon the premature ventricular contraction during His refractoriness, uncorrected/corrected postpacing interval, differential ventricular entrainment, orthodromic His capture, transition zone analysis, and total pacing prematurity. Step 3 characterizes the concealed nodoventricular/nodofascicular pathway and His‐ventricular pathway‐related tachycardia based on observations such as V‐V‐A response, ΔatrioHis interval, and paradoxical reset phenomenon. There is no single diagnostic maneuver that fits all scenarios. Therefore, the ability to apply multiple maneuvers in a case allows the operator to accumulate evidence to make a likely diagnosis. Let's embark on this adventure!

## Graphical abstract

The SVT mechanism includes atrial tachycardia (AT), orthodromic reciprocating tachycardia (ORT) via an atrioventricular accessory pathway (AP), nodoventricular pathway (NVP), nodofascicular pathway (NFP) or His‐ventricular pathway, and atrioventricular nodal reentrant tachycardia (AVNRT). Diagnosis should be made in the following three steps: Step 1 involves differentiating AT from other SVTs based on observations such as V‐A‐V or V‐A‐A‐V response, ΔAA interval, VA linking, the last entrainment sequence, and response to atrial extrastimulus. Step 2 focuses on differentiating ORT from AVNRT based on observations such as tachycardia reset upon His‐refractory PVC, uncorrected/corrected postpacing interval (PPI) − tachycardia cycle length (TCL), differential ventricular entrainment, orthodromic His capture, transition zone analysis, and total pacing prematurity (TPP). Step 3 characterizes ORTs via concealed NV/NFP and HVP‐related mechanisms based on observations such as V‐V‐A response, atrioHis (AH) interval, and paradoxical reset phenomenon.

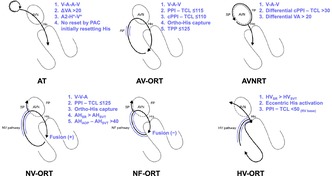

## 1 INTRODUCTION

**FIGURE 1:**
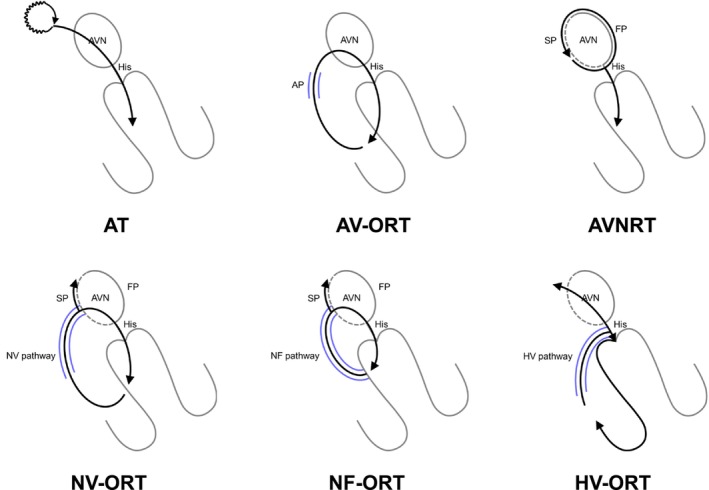
Differential diagnoses of narrow QRS tachycardia include atrial tachycardia (AT), orthodromic reciprocating tachycardia (ORT) via an atrioventricular AP, nodoventricular (NV) pathway, nodofascicular (NF) pathway or His‐ventricular (HV) pathway, and atrioventricular nodal reentrant tachycardia (AVNRT). AP, accessory pathway; AVN, atrioventricular node; FP, fast pathway; SP, slow pathway.

In the field of cardiac electrophysiology, there is a universal desire: the discovery of a flawless diagnostic maneuver for supraventricular tachycardias (SVTs). This is not merely a wish but a shared odyssey. Electrophysiologists face the critical task of differentiating the SVT mechanism, such as atrial tachycardia (AT), orthodromic reciprocating tachycardia (ORT) via an accessory pathway (AP), or atrioventricular nodal reentrant tachycardia (AVNRT) (Figure 1) because the ablation target varies depending on the specific SVT mechanism. In ORT, the primary ablation target is the AP potential or site of the earliest atrial activation. In contrast, for AT, particularly those occurring near the sinus node or atrioventricular node (AVN), the ablation target involves the entrance of the calcium‐sensitive slow conduction zone, which can be determined through entrainment pacing. Furthermore, for AVNRT, there is an established anatomical approach, a pivot point‐guided approach, or a fractionated potential‐guided approach to eliminate the slow pathway (SP).^1^, ^2^, ^3^, ^4^ Although this task may appear straightforward initially, it actually poses a significant challenge. This complexity is largely due to the necessity of mastering a vast array of technical terminology, including terms such as V‐A‐V, V‐A‐A‐V, pseudo‐V‐A‐A‐V, V‐V‐A responses, orthodromic His capture, total pacing prematurity (TPP), A2‐H*‐V*, among others. Additionally, numerous specific cutoff values must be memorized accurately for correct differentiation. Retaining these terms and values, often referenced from prior publications or textbooks, can be a daunting task.

Another complicating factor might be the significant progress in understanding SVT mechanisms in recent years. The mechanism underlying adenosine‐ and verapamil‐sensitive ATs has been identified as reentry.^5^, ^6^ In AVNRT, various variants of the SP, including the superior slow pathway (sup‐SP) located in the anteroseptum and others situated in the inferolateral tricuspid or mitral annulus, have been identified.^7^, ^8^, ^9^ Furthermore, understanding of ORT through an AP has expanded to include the nodoventricular/nodofascicular pathway (NVP/NFP) and His‐ventricular pathway (HVP), in addition to the well‐established atrioventricular AP (Figure 1).^10^ Given these observations, even when tachycardia exhibits an eccentric atrial activation sequence, all three SVT mechanisms are still considered potential candidates for differentiation.

To improve diagnostic accuracy and achieve sufficient sensitivity and specificity, numerous diagnostic maneuvers have been proposed. However, each has its limitations and prompts a search for new diagnostic techniques. This continuous cycle of discovery and refinement, which we titled “SVT Quest” is reviewed in chronological sequence. This adventure in diagnosing narrow QRS tachycardia unfolds in 3 steps: Step 1 involves differentiating AT from ORT and AVNRT; Step 2 focuses on differentiating ORT from AVNRT; and Step 3 characterizes the concealed NVP/NFP and HVP‐related tachycardia. There is no single diagnostic maneuver that fits all scenarios. Therefore, the ability to apply multiple maneuvers in a case allows the operator to accumulate evidence to make a likely diagnosis. Let's embark on this adventure!

### 1.1 Step 1: Differentiating AT from ORT and AVNRT

The first step of the SVT differentiation is distinguishing AT from ORT and AVNRT. AT usually exhibits long RP tachycardia, but still needs to be differentiated from ORT via the AP with the decremental properties or fast‐slow AVNRT, which also exhibit a long RP tachycardia due to the longer conduction time in the retrograde limb (decremental AP/SP) and the relatively short conduction time in the anterograde limb (AVN/FP).

#### 1.1.1 V‐A‐V or V‐A‐A‐V response after right ventricular overdrive pacing

**FIGURE 2:**
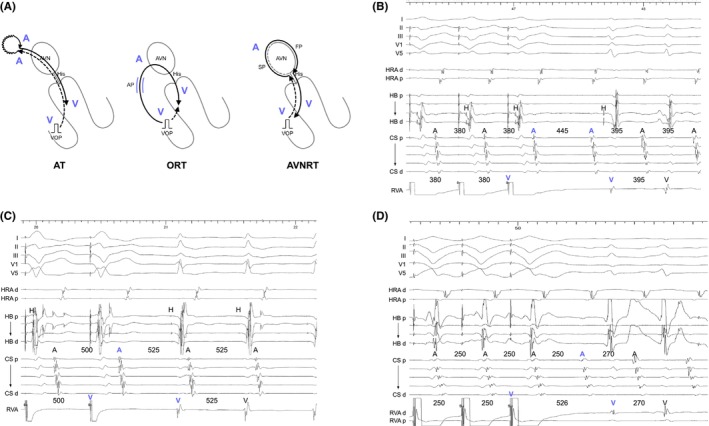
(A) Schematic diagrams depicting the V‐A‐A‐V and V‐A‐V responses after ventricular overdrive pacing (VOP), according to the mechanisms of tachycardia, along with intracardiac electrograms in (B) AT, (C) slow‐slow AVNRT, and (D) fast‐slow AVNRT. A, atrium; AP, accessory pathway; AVN, atrioventricular node; AVNRT; atrioventricular nodal reentrant tachycardia; AT, atrial tachycardia; CS, coronary sinus; d, distal; FP, fast pathway; H, His; HB, His bundle; HRA, high right atrium; ORT, orthodromic reciprocating tachycardia; p, proximal; RVA, right ventricular apex; S, stimulus; SP, slow pathway; VOP, ventricular overdrive pacing; V, ventricle.

Knight et al. were pioneers in establishing diagnostic criteria with right ventricular (RV) overdrive pacing (VOP) for tachycardia.^11^ They found that AT resumes with a V‐A‐A‐V response post‐VOP cessation (Figure 2A,B), while other SVT mechanisms, like ORT and AVNRT, typically resume with a V‐A‐V response (Figure 2A,C).^11^ A key challenge to note is the occurrence of a pseudo‐V‐A‐A‐V response (Figure 2D) but this is merely a visual trick overcome by careful measurement of the last atrial cycle driven at the pacing cycle length (PCL). In cases of ORT via the AP with decremental properties and atypical AVNRT, pacing‐induced decremental conduction through the retrograde AP or SP can cause the paced ventriculoatrial (VA) interval to exceed the PCL, creating a pseudo‐V‐A‐A‐V response. This pseudo‐V‐A‐A‐V response can be identified when the second atrial beat (A2) after VOP accelerates at a PCL.^12^ Thus, for the accurate assessment of a V‐A‐A‐V or V‐A‐V response, electrophysiologists must always determine which atrial electrogram is driven by pacing.

#### 1.1.2 ΔAA interval in the setting of the V‐A‐A‐V response

**FIGURE 3:**
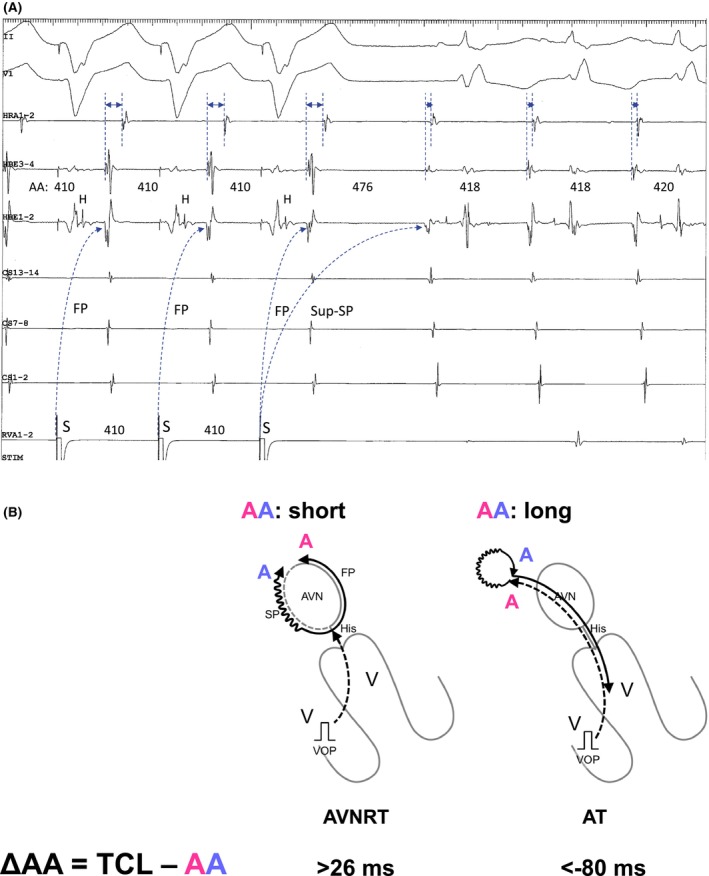
(A) Intracardiac electrograms of a double atrial response utilizing both fast and superior slow pathways in fast‐slow AVNRT via a superior (Sup)‐slow pathway. (B) Schematic diagrams depicting the ΔAA intervals in AVNRT and AT. Abbreviations are as in Figure 2.

However, recent studies have questioned the V‐A‐A‐V response's specificity for AT, revealing that it can also arise from a double atrial response utilizing both fast and slow pathways in AVNRT, notably in fast‐slow AVNRT cases involving either the typical or sup‐SPs (Figure 3A).^13^ These findings indicate that a V‐A‐V response upon resumption of tachycardia effectively rules out AT, whereas a V‐A‐A‐V response requires further assessment due to its occurrence in both AT and AVNRT. Addressing this diagnostic challenge, Kaneko et al. introduced the ΔAA interval to distinguish between V‐A‐A‐V responses in AT and AVNRT attributed to a double atrial response.^13^ The ΔAA interval is determined by subtracting the AA interval during tachycardia (i.e., the tachycardia cycle length, TCL) from the AA interval between the first and second atrial electrograms during the V‐A‐A‐V response. The AA interval during the V‐A‐A‐V response is theoretically shorter in AVNRT because this interval reflects the difference between retrograde FP conduction and SP conduction. In contrast, this interval is theoretically longer in AT because this interval reflects the conduction time of the entire reentrant circuit of AT. A ΔAA interval greater than 26 ms accurately indicates fast‐slowAVNRT with a sensitivity of 76% and a specificity of 100%, while a ΔAA interval less than −80 ms indicates AT with a sensitivity of 50% and a specificity of 100% (Figure 3B). However, a ΔAA interval within the range of −80 to 26 ms still yields an indeterminate diagnosis. Nonetheless, for the vast majority of ATs, the retrograde sequence will change to reflect AVN conduction during VOD, followed by the resumption of the AT sequence.

#### 1.1.3 Differential atrial overdrive pacing

**FIGURE 4:**
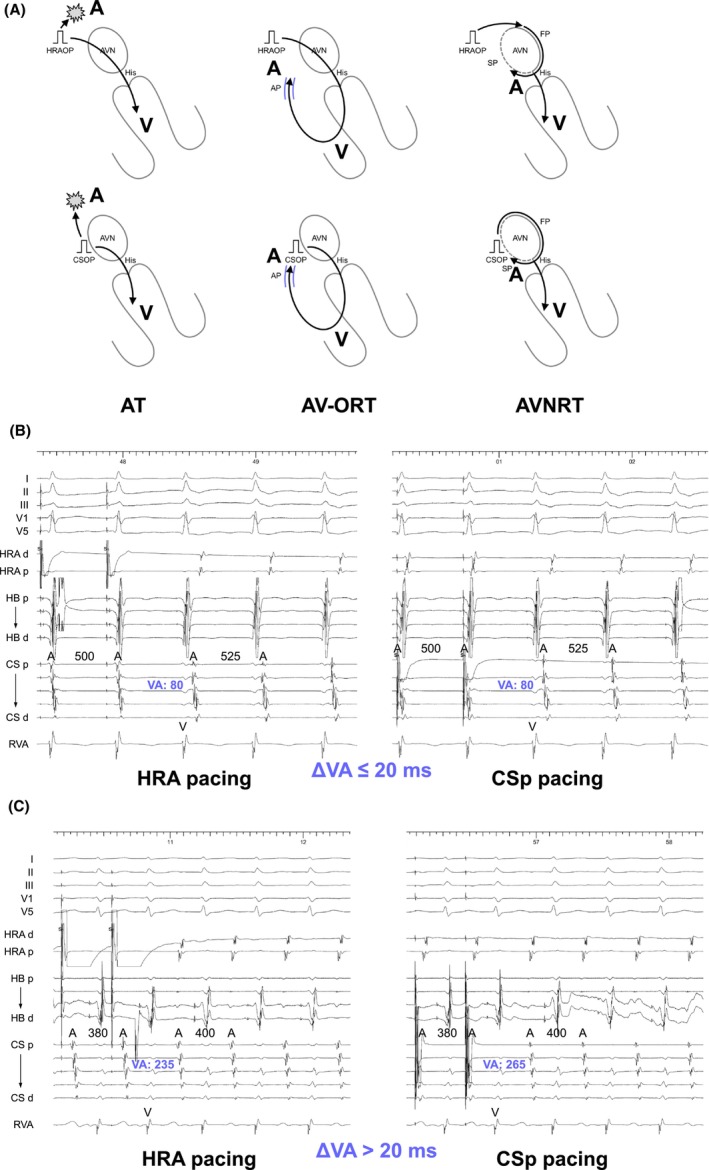
(A) Schematic diagrams depicting the ΔVA interval after atrial overdrive pacing (OP), according to the mechanisms of tachycardia, along with intracardiac electrograms recorded during differential AOP from the high right atrium (left) and proximal coronary sinus (right) in (B) slow‐slow AVNRT and (C) AT. Abbreviations are as in Figure 2.

Another limitation of this method is its dependency on the presence of retrograde VA conduction during VOP; its effectiveness is compromised without consistent 1:1 retrograde VA conduction. To overcome this limitation, Maruyama et al. proposed a diagnostic maneuver that relies on evaluating the VA linking following differential atrial overdrive pacing (AOP). This approach serves as a tool to distinguish AT from other forms of SVTs.^14^ During tachycardia, AOP is applied from three locations—typically the high right atrium (HRA), proximal coronary sinus (CS), and distal CS. The maximal difference in postpacing VA intervals (from the last captured RV electrogram to the earliest atrial electrogram of the first beat after pacing) across these pacing sites is measured as the ΔVA interval (Figure 4A). The ΔVA interval is fixed at ≤14 ms (later modified as 20 ms)^15^ in ORT and AVNRT because the postpacing VA interval reflects the conduction time of the retrograde limb of the tachycardia (AP or AVN), which is identical to the VA interval during the tachycardia (Figure 4B). Conversely, a ΔVA interval >14 ms (later modified as 20 ms)^15^ indicates a lack of VA linking and is diagnostic for AT because the postpacing VA intervals reflect the difference in times between the last atrial pacing capturing the ventricle and the last pacing capturing the AT origin, which varies according to the proximity of the pacing site to AVN and AT origin (Figure 4C). The presence of VA linking excludes AT with 100% sensitivity and specificity.

Maruyama later described the following: originally, a cutoff value of 14 ms for two‐point data was determined. However, the separation was small, although there was no overlap between the groups. Therefore, false positives can arise in AVNRT cases with fluctuating VA intervals. Using three points improves the separation and reduces the possibility of false positives. While the cutoff for three points was set at 20 ms, using 20 ms even for two points makes it more specific and easier to remember. Although false negatives may occur with a cutoff value of 20 ms for two points, assessing a third site with the same 20 ms cutoff improves sensitivity. This is why we modified the delta VA cutoff to 20 ms. Simply put, a ΔVA interval >20 ms with two points is diagnostic for AT, but if it is ≤20 ms, pacing should be considered from another site. If the maximal difference reaches >20 ms, it is diagnostic for AT; if it remains <20 ms, AT can be excluded.

#### 1.1.4 The last entrainment sequence

**FIGURE 5:**
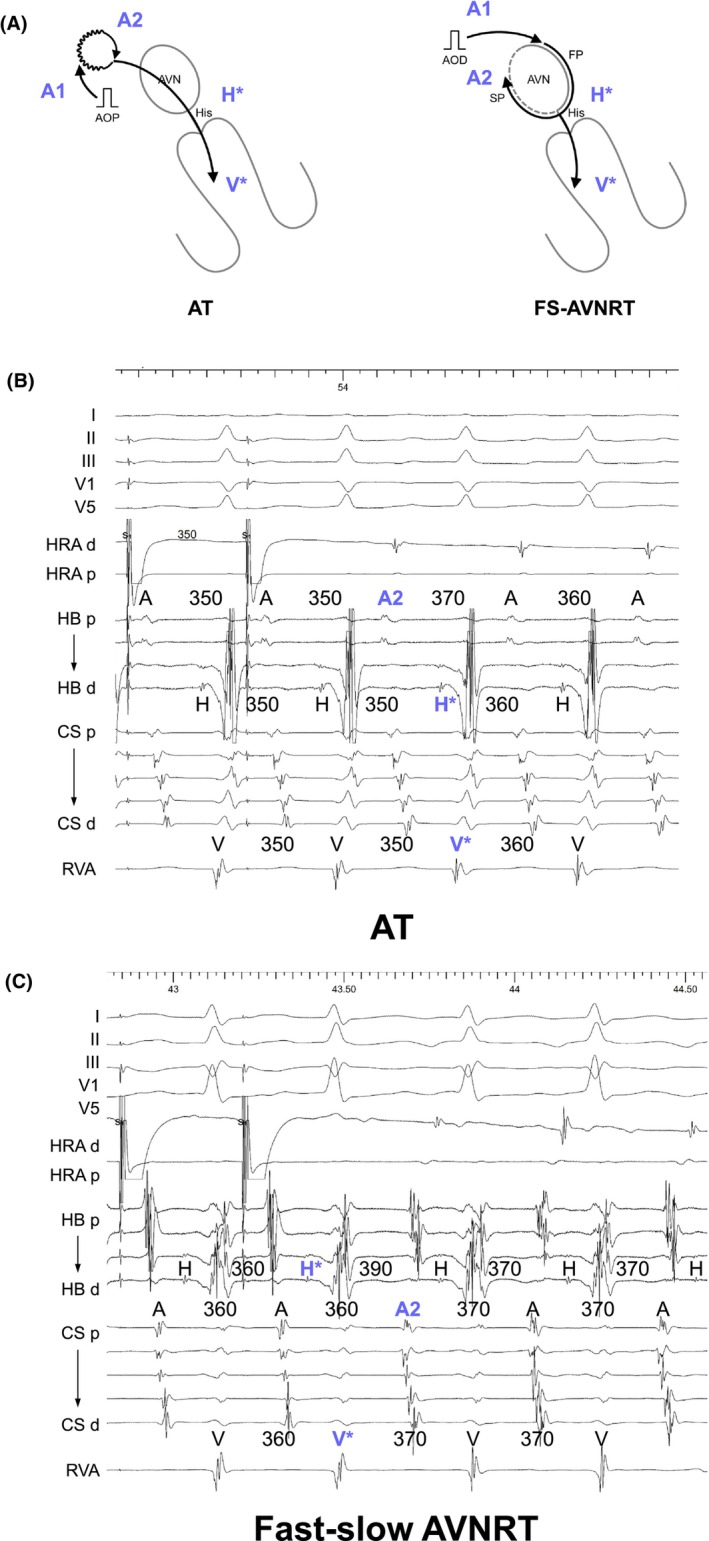
(A) Schematic diagrams depicting the last entrainment sequence after atrial overdrive pacing (AOP), according to the mechanisms of tachycardia, along with intracardiac electrograms recorded during AOP from the high right atrium in (B) paraHisian AT and (C) fast‐slow (FS) AVNRT. Abbreviations are as in Figure 2.

However, later research showed that fast‐slow AVNRTs involving either the typical or sup‐SPs, frequently exhibit a lack of VA linking.^16^ They explained that the variation in the VA interval after AOP from different sites was due to the pacing site‐dependent effect on retrograde conduction time over the SP in the first beat following AOP cessation. This occurs because the depth of antidromic penetration into the SP varies depending on the proximity of the pacing site to the AV node. To address this issue, Maruyama et al. subsequently suggested that analyzing the last entrainment sequence after AOP could accurately diagnose reentrant AT.^15^ The initial step involves measuring A1–A2 at the earliest atrial activation site (EAAS) after AOP cessation. Orthodromic capture is verified if A2 accelerates to the (PCL). Conversely, antidromic capture is indicated if A1, but not A2, accelerates to the PCL. In the presence of antidromic capture across all recorded atrial electrograms, AOP is repeated from another atrial site until orthodromic capture at the EAAS is achieved. Following confirmation of orthodromic capture, the sequence of A2, and the last His (H*) and RV (V*) electrograms accelerated to the PCL, is analyzed (Figure 5A). The A2‐H*‐V* response is diagnostic of AT, with an 84% sensitivity and 100% specificity (Figure 5B). Conversely, H*‐V*‐A2 and H*‐A2‐V* suggest ORT or AVNRT (Figure 5C). However, a false‐negative or pseudo‐H*‐V*‐A2 response may occur in cases of tricuspid annular AT, particularly when the AOP site is nearer to the AV node than to the AT circuit. A pseudo‐H*‐V*‐A2 response might result when the AV node is situated between the pacing site and the AT circuit, potentially leading to incorrect targeting of the slow pathway. The advantage of this diagnostic approach lies not only in its accuracy for AT diagnosis but also in its implications for ablation strategy, especially for para‐Hisian or perinodal AT. The site orthodromically captured by AOP indicates that the pacing site is proximal to the slow conduction zone of the reentrant AT circuit, providing essential information for a safer ablation strategy known as Yamabe's method.^5^, ^6^ Upon orthodromic capture of the EAAS, radiofrequency energy can be applied starting 2 cm from the EAAS towards the pacing site, progressively moving closer until AT termination.

#### 1.1.5 A single atrial extrastimulus initially resetting the His electrogram

**FIGURE 6:**
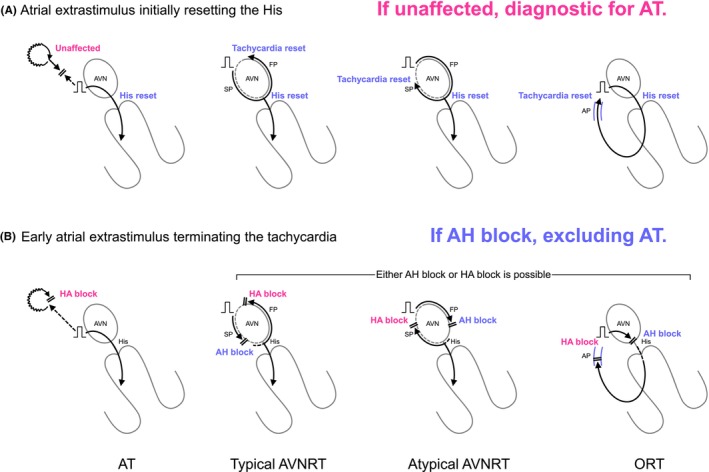
Schematic diagrams depicting (A) the atrial extrastimulus initially resetting the His and (B) the early atrial extrastimulus terminating the tachycardia, according to the mechanisms of tachycardia. AH, atrioHis; HA, His‐atrial. Other abbreviations are as in Figure 1.

The utility of these diagnostic maneuvers remains limited if the tachycardia terminates or if sustained 1:1 AV conduction cannot be achieved during AOP, rendering the assessment indeterminate. To overcome this challenge, Inaba et al. developed new diagnostic criteria using a single atrial extrastimulus.^17^, ^18^ This extrastimulus is applied in the proximal CS, with the coupling interval gradually decreased until it first resets the His bundle, and is continued until tachycardia termination. Tachycardia reset is identified by measuring the atrial interval over two cycles surrounding the extrastimulus. The lack of tachycardia reset immediately following an atrial extrastimulus that initially resets the His electrogram confirms AT with 96% sensitivity and 100% specificity (Figure 6A).^18^ Additionally, tachycardia termination via an AH block induced by the atrial extrastimulus excludes AT with 92% sensitivity and 100% specificity (Figure 6B).^18^ This approach offers some advantages, such as a lower risk of tachycardia termination compared to overdrive pacing, which is a common pitfall in assessing V‐A‐V or V‐A‐A‐V responses during VOP, and VA linking and last entrainment sequence during AOP. It remains more feasible than VOP or AOP even if the tachycardia is terminated by the atrial extrastimulus, and is suitable for SVTs with 2:1 AV conduction. However, limitations exist, including uncertain assessments for tachycardias with fluctuating cycle lengths, although termination still provides diagnostic insights. There is a 4% risk of false negatives where an extrastimulus in the proximal CS might reset both the His electrogram and AT simultaneously.

In light of these diagnostic approaches, it is recommended to initially perform VOP. A V‐A‐V response excludes AT, facilitating further differentiation between ORT and AVNRT using the same electrogram tracing. However, if a V‐A‐A‐V response is observed, there remains a small possibility of a double atrial response in AVNRT, necessitating further assessment of the ΔAA interval in the tracing. If the retrograde activation sequence is the same as the VAAV then it is either AT with an identical sequence to retrograde conduction through the AVN (never seen this) or unusual AP or SP. In situations where consistent 1:1 retrograde VA conduction is not achieved, differential AOP should be employed to evaluate VA linking and the last entrainment sequence. Additionally, if tachycardia terminates during VOD or AOD, an atrial extrastimulus is recommended. The coupling interval should be progressively shortened until it initially resets the His bundle, continuing until the cessation of tachycardia.

### 1.2 Step 2: Differentiating ORT from AVNRT

After ruling out AT, the next step is to differentiate between ORT and AVNRT. Electrophysiologists should be aware that since all current diagnostic maneuvers are designed to distinguish ORT from AVNRT, there exists a small risk of underdiagnosing ORT if these maneuvers yield inconclusive results. It is crucial to understand that AVNRT can only be definitively diagnosed after ORT has been excluded using all diagnostic criteria. Distinguishing between ORT through a septal pathway and atypical AVNRT presented significant challenges in SVT differentiation. ORT, facilitated by the free wall AP, demonstrated eccentric atrial activation, a distinctive feature that sets it apart from AVNRT (although this feature cannot currently rule out atypical AVNRT with SP variants). Conversely, slow‐fast AVNRT is characterized by a His‐atrial (HA) interval ≤70 ms, a criterion that excludes ORT. From this perspective, diagnostic maneuvers have been meticulously developed to differentiate septal ORT from atypical AVNRT. Organizing these maneuvers in chronological order helps in more easily understanding their advantages, importance, and limitations.

#### 1.2.1 Para‐Hisian pacing

Before the diagnostic maneuvers during the SVT, para‐Hisian pacing should be introduced. Hirao et al. pioneered this maneuver to determine the presence of retrograde AP conduction during ventricular pacing.^19^ Para‐Hisian pacing is performed adjacent to the His bundle and proximal right bundle (RB), initially at high output to capture both His and RV (H + Vc). The output is then gradually reduced until loss of His capture (Vc), which is confirmed by the widening of the QRS complex or appearance of the His potential with prolongation of the ventricular‐His interval.

**FIGURE 7:**
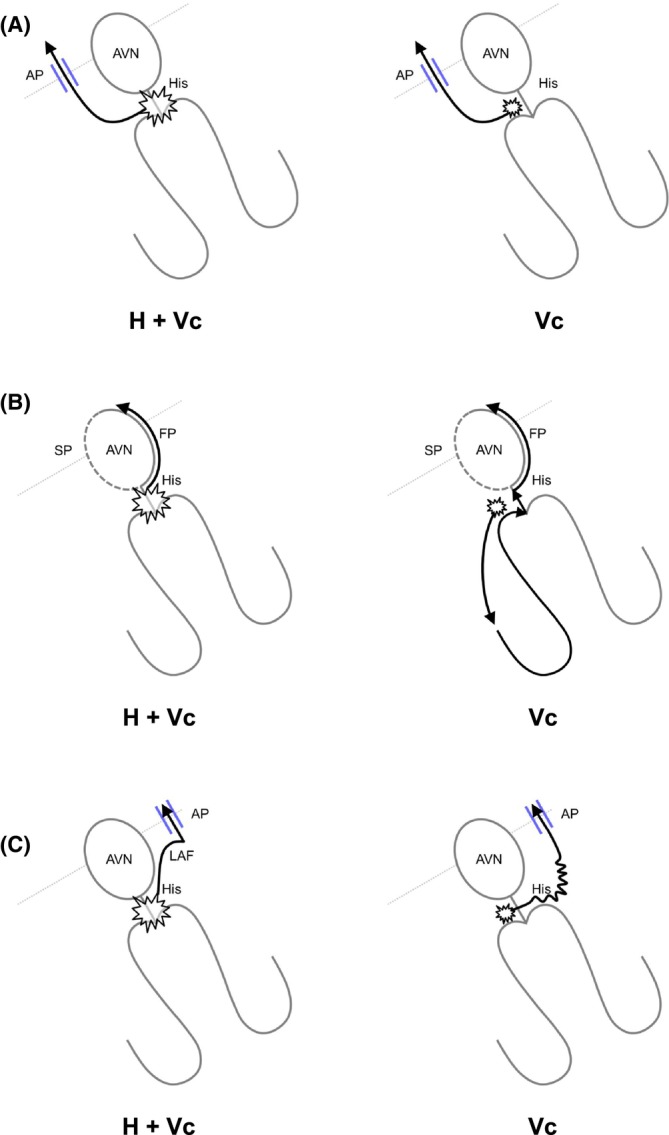
Conduction diagrams during para‐Hisian pacing depicting pacing for His and para‐Hisian ventricular myocardium capture (H + Vc, left) and ventricular capture (Vc, right), explaining (A) AP/AP pattern, (B) AVN/AVN pattern, and (C) AP/AP_L_ pattern. Abbreviations are as in Figure 1.

As a first step, the change in the sequence of retrograde atrial activation between H + Vc and Vc is assessed. Identical atrial activation sequence between H + Vc and Vc indicates exclusive retrograde AP or AVN conduction, whereas a change in atrial activation sequence indicates the presence of both retrograde AP and AVN conductions. As a second step in the setting of exclusive retrograde conduction, the change in stimulus‐atrial (SA) interval is assessed. Identical SA interval indicates exclusive retrograde AP conduction (AP/AP pattern, Figure 7A); retrograde conduction is dependent on local ventricular activation and not on His bundle activation. In contrast, an increase in SA interval without change in HA interval upon Vc indicates exclusive retrograde AVN conduction (AVN/AVN pattern, Figure 7B); retrograde conduction is dependent on His bundle activation and not on local ventricular activation. Despite SA interval prolongation upon V capture, if this is attributed mainly to the prolongation of the S‐local ventricular interval and similar local VA interval close to the AP conduction, it indicates exclusive retrograde AP conduction (AP/AP_L_ pattern, referring to “lengthening,” Figure 7C). Two plausible mechanisms can be explained for the delay in the timing of ventricular activation close to the AP. First, activation over the His‐Purkinje system results in earlier ventricular activation near the AP located distant from the para‐Hisian area. Second, decreasing the pacing output for Vc results in a small delay in ventricular activation in the para‐Hisian area. A recent report determines the cutoff values for exclusive AP and AVN conductions: an SA interval prolongation in pCS of <37 ms or in HRA of <32 ms indicates the presence of a septal AP, whereas a prolongation in pCS of >51 ms or in HRA of >75 ms excludes the septal AP.^20^

However, several caveats and limitations should be noted. First, inadvertent atrial capture should be considered when the SA interval is <60 ms in the electrograms of the pCS and <70 ms in the HRA.^21^ Furthermore, para‐Hisian pacing poses a risk of failing to identify the retrograde conduction of left free wall APs and septal APs with decremental properties, due to the longer time required to reach the atrium via the APs compared to the AVN. Therefore, not only the location but also the conductivity of the AP should be accounted for. Additionally, this technique is limited in patients with proximal RB branch block. Last, the identification of retrograde AP conduction does not necessarily mean that the diagnosis is ORT. The diagnosis of AVNRT via a bystander AP is also possible.^22^ Therefore, an accurate diagnosis requires the diagnostic maneuver during the SVT described below.

#### 1.2.2 A scanned single ventricular extrastimulus during the His‐bundle refractoriness

**FIGURE 8:**
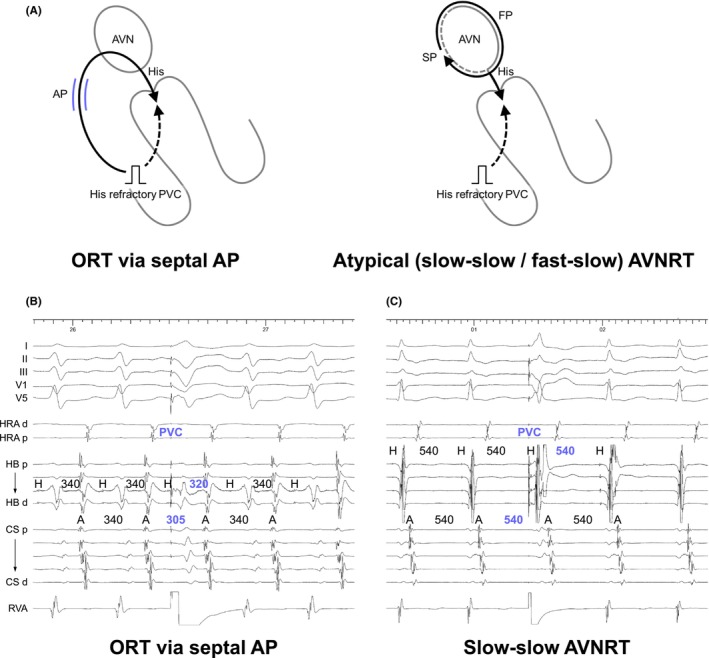
(A) Schematic diagrams depicting a scanned single PVC delivered during His‐refractoriness, according to the mechanisms of tachycardia, along with intracardiac electrograms in (B) ORT via a septal AP and (C) slow‐slow AVNRT. Abbreviations are as in Figure 2.

This maneuver was originally introduced by Wellens in 1967.^23^ The term “premature ventricular contraction (PVC) during His‐refractoriness” is also currently utilized. A single extrastimulus is delivered via the RV catheter, and the coupling interval is systematically reduced by 5–10 ms until the pacing spike either overlaps or precedes the His potential by 20–25 ms. If tachycardia is reset ≥10 ms following pacing, regardless of whether it is advanced or delayed, it is indicative of ORT (Figure 8A,B). Similarly, ORT can be diagnosed if tachycardia terminates without atrial capture, as this indicates a conduction block over the AP. It is important to note that electrophysiologists differentiate between the resetting of the His interval straddling the PVC, referred to as tachycardia reset, and the resetting of the atrial interval straddling the PVC, known as local reset. A reset in the atrial interval without a corresponding reset in the His interval suggests the AP may be a bystander in the tachycardia circuit, though this is not definitive as it could result from a compensatory delay in the AVN following atrial advancement. In contrast, AVNRT cannot be reset by the His‐refractory PVC (Figure 8A,C). Furthermore, the tachycardia can be reset or terminated without atrial capture, even one cycle following a His‐refractory PVC. This phenomenon is specifically diagnostic of AVNRT with a bystander cNVP, which will be described in further detail in the NV pathway section.

#### 1.2.3 Postpacing interval − tachycardia cycle length after RV overdrove pacing

**FIGURE 9:**
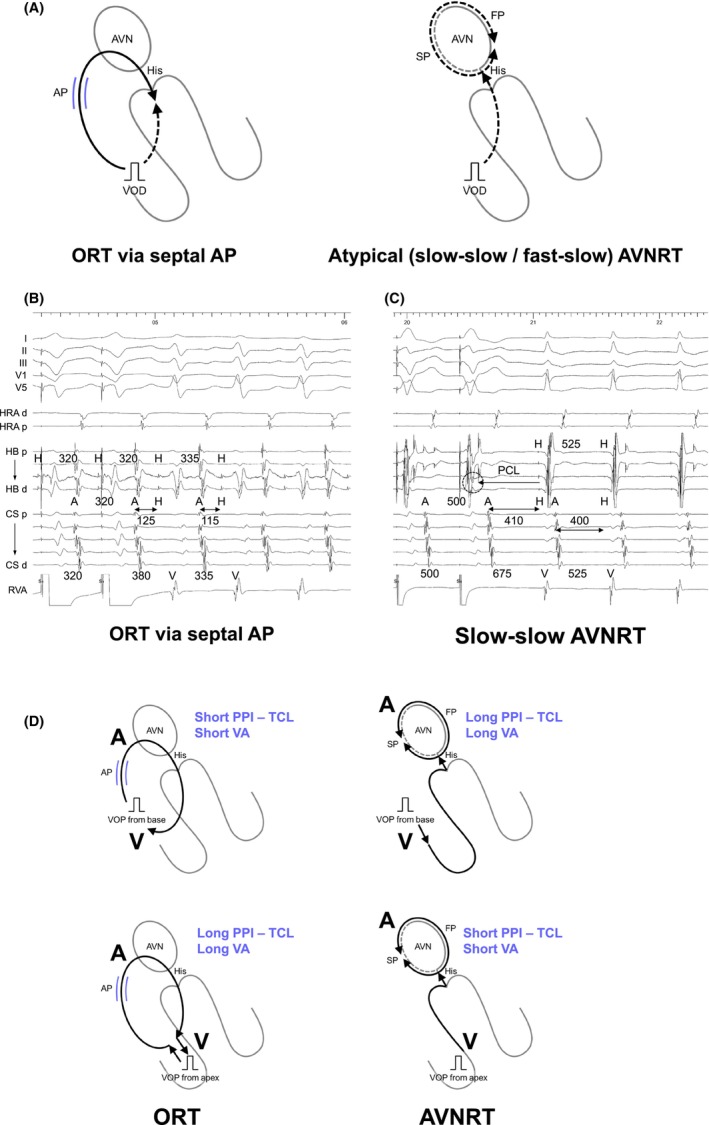
(A) Schematic diagrams depicting ventricular overdrive pacing (VOP), according to the mechanisms of tachycardia, along with intracardiac electrograms in (B) ORT via a septal AP and (C) slow‐slow AVNRT. (D) Schematic diagrams depicting differential ventricular entrainment; VOD from right ventricular base (upper panel) and right ventricular apex (lower panel). PCL, pacing cycle length. Other abbreviations are as in Figure 2.

The assessment of resetting following His‐refractory PVC remains uncertain for tachycardias with spontaneously fluctuating cycle lengths, although termination still provides diagnostic insights. Recognizing these challenges, VOD has emerged as a key tool in diagnosing ORT since 2001. Michaud was the pioneer in applying a measurement‐based diagnostic approach in VOD, emphasizing the significance of the postpacing interval (PPI).^24^ The PPI minus the tachycardia cycle length (TCL) essentially represents double the conduction time through the RV pacing site and the tachycardia circuit. A cutoff value of PPI − TCL ≤115 ms distinctly identifies ORT from AVNRT, given the larger size of the ORT circuit involving both the ventricle and atrium, and its proximity to the RV pacing site in contrast to the AVNRT circuit, which is confined to the AVN (Figure 9A–C). Following a similar concept, the SA interval minus the VA interval was calculated. The SA interval was determined by measuring from the last pacing spike to the last captured electrogram in the HRA during VOD, whereas the VA interval was gauged from the onset of the QRS complex to the HRA electrogram. A cutoff value of SA − VA ≤85 ms effectively distinguishes ORT from AVNRT. This study offers critical insights, particularly concerning patients with septal ORT and atypical AVNRT, while excluding those with free wall AP and typical slow‐fast AVNRT, addressing the major concerns.

Note that for accurate measurement avoiding SVT termination, the PCL of VOD should be set as close as possible to the TCL (or shortest TCL), and VOD should be synchronized to start at the timing when it overlaps with the RV electrogram. Furthermore, another tip is that PPI − TCL can be measured during tachycardia induction and upon a scanned PVC, and can be refined by pacing near the site of earliest atrial activation.

#### 1.2.4 Corrected postpacing interval − tachycardia cycle length

The PPI − TCL is considered an excellent tool for differentiating ORT from AVNRT, yet it has significant limitations. For successful entrainment of the tachycardia circuit through VOD, the PCL must be set 10–30 ms shorter than the TCL. As a result, the PPI may be prolonged due to decremental conduction properties, raising the risk that ORT might be underdiagnosed due to a prolonged PPI. To overcome this challenge, González‐Torrecilla introduced the corrected PPI − TCL, which compensates for the delay in AVN conduction time during VOD.^25^ In practice, this delay can be calculated by taking the postpacing atrial‐His (AH) interval—measured from the last captured electrogram in the HRA to the His electrogram in the first return beat after VOD—and subtracting the AH interval observed during the tachycardia. While the uncorrected PPI − TCL and the SA − VA interval present considerable overlaps among septal ORT, free wall ORT, and AVNRT, particularly when pacing much faster than the TCL, a corrected PPI − TCL ≤110 ms effectively distinguishes ORT from AVNRT (Figure 9B,C). Nonetheless, this correction proves useful only when the PPI – TCL is calculated to be >115 ms, to avoid the underdiagnosis of ORT. This correction is unnecessary when PPI − TCL is ≤115 ms, as in these instances, the PPI − TCL alone can accurately diagnose ORT.

#### 1.2.5 Differential ventricular entrainment

Uncorrected/corrected PPI − TCL appears to be a robust method but is limited by its reliance on a single absolute value (PPI from the RV apex), making it susceptible to problematic borderline values, which are not uncommon. These cases are likely related to differences in RV conduction time and the exact location of the RV apical catheter. Addressing this challenge, Segal et al. introduced differential ventricular entrainment to compare the PPI and VA (=SA) interval between pacing from the His catheter (RV base) and pacing from the RV catheter (RV apex).^26^ Differential corrected PPI − TCL and VA (=SA) intervals are calculated by subtracting these values as (RV base − RV apex). This method is based on an early study of the differential VA interval (RV base − RV apex) during sinus rhythm; the differential VA interval is negative in the presence of an AP and positive in its absence. Because the AP is located in the basal area, the VA interval during pacing from the RV base is shorter in the presence of an AP, while the VA interval during pacing from the RV apex is shorter in the absence of an AP due to the proximity of the RV apex to the His‐Purkinje system, which conducts to the AVN.^27^

Similarly, during ORT, the corrected PPI − TCL and VA (=SA) intervals are shorter in VOP from the RV base because the AP is located in the basal area, resulting in the circuit being closer to the RV base compared to the RV apex (Figure 9D). Conversely, during AVNRT, the RV apex is closer to the His‐Purkinje system, which conducts to the AVN circuit (Figure 9D). The differential cPPI − TCL >30 ms or differential VA interval >20 ms is diagnostic of AVNRT with a sensitivity and specificity of 100%. The advantage of this method is the sufficient gaps in these values between ORT and AVNRT, even including free wall ORT. However, the diagnostic performance may be insufficient due to the small sample size and the lack of cases with an AP with decremental properties, which is the most challenging differentiation as described in the next section.

#### 1.2.6 Orthodromic His/septal ventricular capture during VOP

The corrected PPI − TCL is recognized as a valuable tool for distinguishing ORT from AVNRT, yet it still faces significant limitations. One such limitation is the decremental conduction observed not only in the AVN but also in septal APs. This can lead to a prolonged PPI due to the decremental conduction properties in the septal AP, even after correction for the AH interval. This raises the possibility of underdiagnosing ORT. Addressing the limitations of measurement‐based evaluations, Nagashima and Michaud explored the direction of His bundle activation during VOD.^28^ During VOD, the His bundle and/or ventricular septum can be activated in an anterograde direction, indicating orthodromic capture of the His bundle/ventricular septum by VOD (Figure 9A). This observation is explained by the orthodromic wavefront conducting retrogradely through the AP and then anterogradely through the AVN and His bundle, where it collides with the antidromic wavefront from the subsequent pacing cycle. Stable fusion below the His bundle, marked by consistent QRS fusion, occurs once the wavefronts equilibrate. Upon cessation of pacing, the His bundle is activated next at the PCL, driven by the last paced wavefront traveling retrogradely through the pathway and anterogradely through the AVN (Figure 9B). This maneuver allows for a straightforward and rapid assessment, as orthodromic His/septal ventricular capture can be determined by measuring from the His bundle or ventricular electrogram back an interval equal to the PCL. Identification of the preceding His bundle or ventricular electrogram at this interval indicates orthodromic capture. Conversely, in AVNRT, the His bundle is always activated retrogradely (antidromic His capture) to entrain the AVN circuit (Figure 9A). Thus, no His signal is observed at this interval (Figure 9C). The retrograde His bundle electrogram is often obscured by overlapping ventricular electrograms, making retrograde capture of His bundle inferred by the clear absence of anterograde His capture. This maneuver accurately diagnoses ORT in 60% of cases via a slowly conducting AP.

#### 1.2.7 Transition zone analysis

**FIGURE 10:**
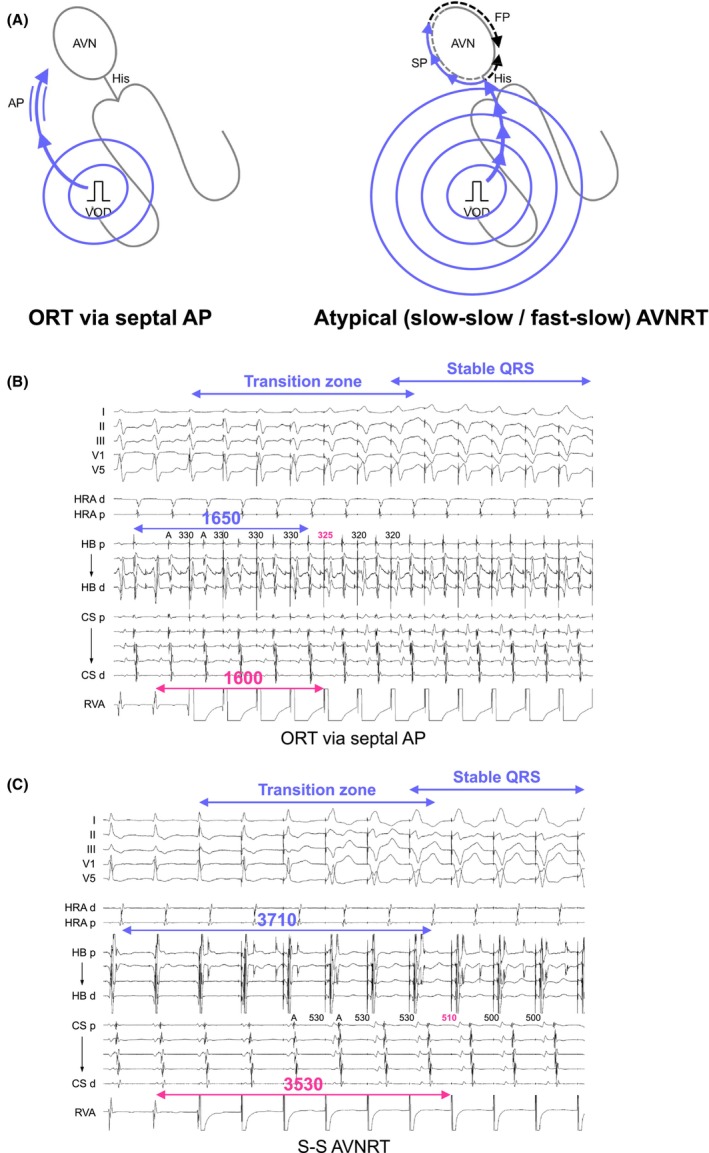
(A) Schematic diagrams depicting the initiation of ventricular overdrive pacing (VOP) until the pacing captures the atrium, according to the mechanisms of tachycardia, along with intracardiac electrograms in (B) ORT via a septal AP and (C) slow‐slow AVNRT. Abbreviations are as in Figure 2.

Another challenge with VOP is the termination of tachycardia, which can make the assessment uncertain. In response to this, AlMahameed and Michaud introduced an approach focusing on the initiation of the VOP.^29^ This method, grounded in VOP, leverages the principle of resetting ORT through the application of His‐refractory PVCs. They defined a “transition zone” as the period during which paced complexes exhibit progressive QRS fusion up to the first paced complex that shows stable QRS morphology, as determined through a 12‐lead ECG analysis. In 94% of ORT cases, atrial timing perturbations observed within the transition zone—including either advancement or delay of ≥15 ms, and termination with VA block—are indicative of VOP capturing the AVN or AP (Figure 10A,B). However, 6% of ORTs showed atrial advancement of only 10 ms which was possibly due to the PCL is shorter than TCL by <15 ms. Whereas, in 97% of ORT cases, the SA interval becomes fixed, showing variations of <10 ms during the transition zone, and all ORT patients exhibit either atrial timing perturbation or a fixed SA interval. Nonetheless, it is recommended that the PCL be 20–40 ms shorter than the TCL in this maneuver. Conversely, atrial timing perturbations and the fixed SA interval manifest after the transition zone in AVNRT (Figure 10A,C). The greatest advantage of this diagnostic maneuver is the feasibility of the assessment regardless of tachycardia termination during the VOP. However, the assessment based on a 12‐lead ECG lacks objectivity. Furthermore, a pitfall is that the diagnostic performance is insufficient when the atrial perturbation occurs in the first beat showing stable QRS morphology because this could occur in either ORT or AVNRT.

#### 1.2.8 Total pacing prematurity

To enhance the diagnostic accuracy of transition zone analyses and address the issue of subjectivity, Maruyama et al. introduced the concept of TPP. This approach quantifies the pacing‐induced prematurity necessary to initially reset or terminate the tachycardia (Figure 10A). TPP is defined as the cumulative prematurity of each stimulus (TCL − PCL) up to the point of the first atrial resetting or tachycardia termination. This is calculated by subtracting the PCL from the TCL and then multiplying it by the number of stimuli (*n*) required to achieve atrial resetting or tachycardia cessation, as illustrated by the following formula.

$$\mathrm{TPP}=n\left(\mathrm{TCL}-\mathrm{PCL}\right)=n\mathrm{TCL}-n\mathrm{PCL}$$

A cutoff value of TPP <125 ms is diagnostic for ORT, yielding excellent diagnostic accuracy (Figure 10B,C). Additionally, TPP can be measured even if tachycardia terminates during VOP. Furthermore, because the process of entraining the tachycardia circuit is similar yet opposite to the response after cessation of VOP, PPI − TCL can be predicted by deducting the tachycardia advancement during the initial atrial reset from the TPP as illustrated by the following formula.

PredictedPPI−TCL=TPP−tachycardia advancement.

This maneuver yields satisfactory diagnostic accuracy through more objective and quantifiable means. However, the accuracy of this maneuver has not yet been sufficiently verified for ORT via decremental AP, and particularly when pacing much faster than the TCL.

### 1.3 Step 3: Concealed NV/NF pathway and His‐ventricular pathway‐related tachycardia

It appears these diagnostic maneuvers had brought an end to the entire adventure of diagnosing SVT. However, accompanied by histological clarification, the understanding of ORT through an NV/NF pathway has been clarified. Diagnosing ORT via the rare NVP/NFP has become the most recent, challenging differentiation. Recently, 2 diagnostic criteria have been introduced from different perspectives: one for differentiating NV/NF‐ORT from AVNRT,^10^ and the other for differentiating NV/NF‐ORT from ORT via atrioventricular AP with decremental properties.^30^, ^31^

#### 1.3.1 Differentiating NV/NF‐ORT from AVNRT

**FIGURE 11:**
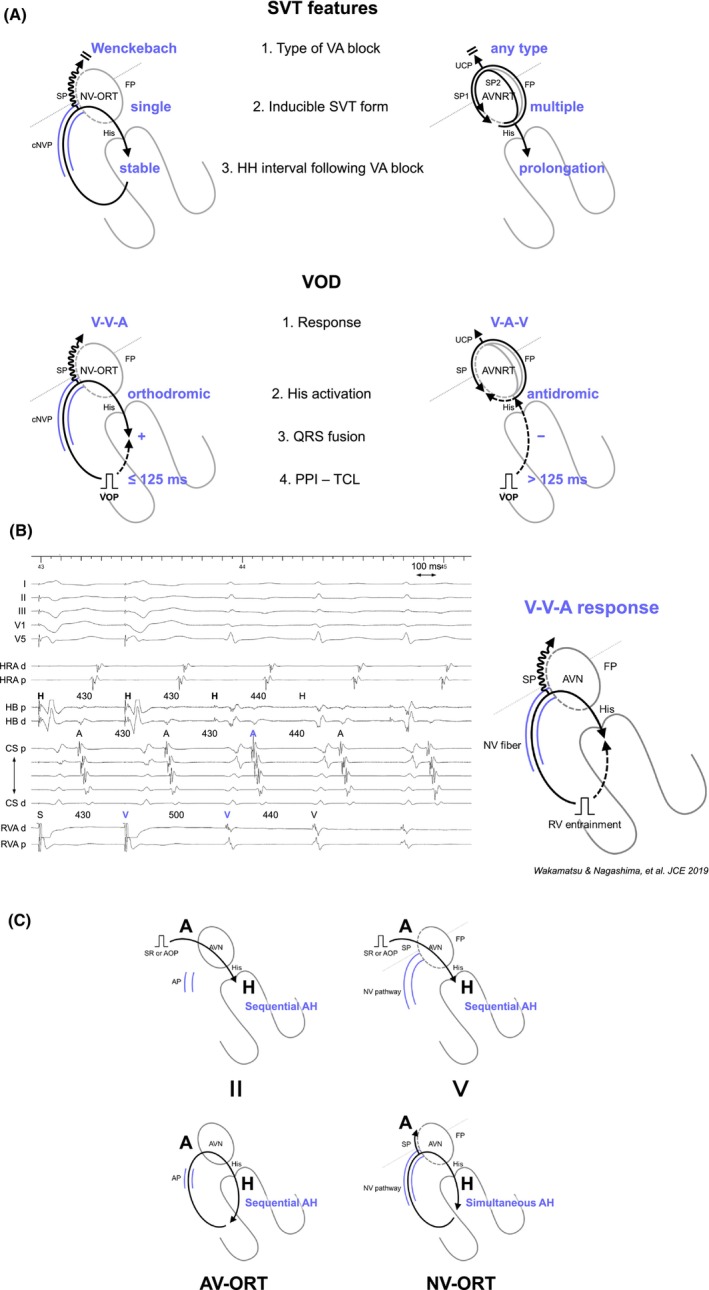
(A) Schematic diagrams showing the tachycardia features and responses during ventricular overdrive pacing (VOD) for the ORT via a concealed nodoventricular pathway (cNVP) and AVNRT with an upper common pathway (UCP), along with intracardiac electrograms in (B) NV‐ORT. (C) Schematic diagrams depicting AH interval during sinus rhythm (SR) or atrial overdrive pacing (AOP, upper panel), and tachycardia in ORT via an atrioventricular accessory pathway (AP) and via cNVP (lower panel). Abbreviations are as in Figure 2.

Because the circuit of NV/NF‐related ORT does not involve the atrium, and the atrium is typically activated through retrograde conduction over the SP—which is commonly connected to the NV/NFP—the first step in recognizing the presence of a concealed NV/NFP is the persistence of SVT despite the occurrence of a VA block. From this standpoint, NV/NF‐ORT must be distinguished from AVNRT, especially in the presence of an upper common pathway block. SVT with VA block suggests that the atrium is neither the origin nor a part of the tachycardia circuit, thereby excluding AT and ORT via atrioventricular AP. Nonetheless, a careful interpretation of intracardiac electrograms is essential to differentiate between NV/NF‐ORT and AVNRT. Nagashima and all Japan coauthors (EP University members described in acknowledgment) developed diagnostic criteria (Figure 11A) including: (1) a V‐V‐A response due to an extremely long SA interval exceeding the TCL and (2) orthodromic His capture during VOD, as indicative of NV/NF‐ORT (Figure 11B).^10^, ^32^ Moreover, (3) a PPI − TCL ≤125 ms and the presence of QRS fusion help to distinguish NV‐ORT from NF‐ORT and AVNRT. Additionally, observations such as (1) a single form of inducible SVT versus multiple SVTs with varying VA intervals, (2) a Wenckebach‐type VA block during tachycardia vs a non‐Wenckebach type, and (3) a stable His interval despite VA block versus fluctuating TCL straddling the VA block, suggest NV/NF‐ORT versus AVNRT.^33^

#### 1.3.2 Differentiating NV/NF‐ORT from ORT via atrioventricular AP with decremental properties

In the situation where AVNRT is excluded by tachycardia or atrial reset following His‐refractory PVC, or within the transition zone during RVOP, the most challenging differentiation is between NV/NF‐ORT and ORT via atrioventricular AP with decremental properties. Although these tachycardias share an identical lower limb (His‐Purkinje system/ventricle), their upper limb differs: AVN in NV/NF‐ORT versus the atrium in atrioventricular‐ORT. Therefore, considering these distinctions, unlike atrioventricular‐ORT, in NV/NF‐ORT the atrium is not part of the circuit. Ho et al. introduced diagnostic criteria to differentiate NV/NF‐ORT from ORT via atrioventricular AP with decremental properties: (1) VA block during tachycardia, (2) VA variability despite a constant TCL as proof that the atrium is downstream of the circuit, (3) AH <40 ms (an interval too short to represent true AV nodal conduction) during tachycardia, (4) AH_SR_ > AH_SVT_ (a paradoxical response to the normal decremental properties of the AVN, Figure 11C), and (5) ΔAH: AH_atrial entrainment/pacing_ − AH_SVT_ >40 ms (indicating sequential vs. simultaneous AVN activation), due to the AH interval being a pseudo‐interval (simultaneous AH activation) during NV/NF‐ORT, as opposed to the true intervals during atrioventricular‐ORT (sequential AH activation, Figure 11C).^30^

#### 1.3.3 Para‐Hisian pacing in the setting of NV/NFP

Theoretically, during para‐Hisan pacing with conduction only over a nodal pathway, an NVP would show an AP/AP response because conduction is dependent upon myocardial capture. By contrast, an NFP is dependent upon fascicular capture and therefore, the SA interval would be shorter with than without His‐proximal RB bundle capture. However, the HA interval would be shorter with a more distal RB insertion (akin to AVN/AP response but without change in the atrial activation pattern) and unchanged with a more proximal RB insertion (akin to AVN/AVN response). If, however, retrograde conduction occurs over both the nodal pathway and the His‐AV node limb, then the responses are more complex and depend upon which structure reaches the AVN first with and without His‐proximal RB capture. Nagashima et al. observed varying responses to para‐Hisian pacing in the context of an NVP, which demonstrated different responses (AVN/AVN, AVN/AP, or AP/APL), potentially depending on the NVP's conduction time.^34^ Whereas, in the context of an NFP, responses typically follow the AVN/AVN response. However, the diagnosis of an NVP was established by the presence of QRS fusion during resetting/entrainment but this concept has not been truly validated. Theoretically, an NFP can demonstrate QRS fusion during resetting/entrainment if the collision point between orthodromic and antidromic wavefronts is in the RB (between the His bundle and insertion of the NFP). In this case, orthodromic wavefronts leaving the left His‐Purkinje system can collide with paced antidromic wavefronts from the RV giving rise to paced QRS fusion. This is particularly true with a more distal NF insertion where antidromic penetration of the right‐sided Purkinje system does not need to be deep to penetrate the NF‐ORT circuit. Based on this discussion, para‐Hisian pacing is a potentially useful maneuver to differentiate NVP from NFP, in addition to identifying fusion during RV entrainment pacing.

#### 1.3.4 AVNRT with a bystander NV/NFP

**FIGURE 12:**
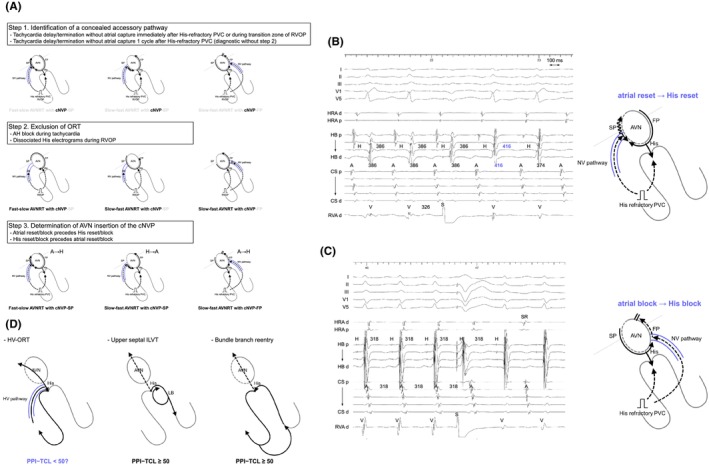
(A) Schematic diagrams of the 3 steps to diagnosing AVNRT with a bystander concealed nodoventricular (NV) pathway. Intracardiac electrograms and the schematic diagram in (B) fast‐slow AVNRT with a bystander NV pathway connecting to the slow pathway, and (C) slow‐fast AVNRT with a bystander NV pathway connecting to the fast pathway. (D) Schematic diagrams of orthodromic reciprocating tachycardia via the His‐ventricular pathway (HV‐ORT), upper septal type of idiopathic left ventricular tachycardia (ILVT), and bundle branch reentry. PVC, premature ventricular contraction; PPI, postpacing interval; TCL, tachycardia cycle length. Other abbreviations are as in Figure 2.

In developing these diagnostic criteria for NV/NF‐ORT, it has become apparent that the differential diagnosis is not necessarily exclusive to either ORT or AVNRT. There have been several reports of AVNRT with a bystander concealed NVP.^10^, ^30^, ^35^, ^36^, ^37^, ^38^, ^39^, ^40^, ^41^, ^42^, ^43^, ^44^ Nagashima and all Japan coauthors (EP University members) developed comprehensive diagnostic criteria for AVNRT with a bystander concealed NVP (Figure 12A).^45^ The diagnosis is made through the following three steps.

Step 1 involves identifying the presence of a concealed AP with ventricular insertion. The resetting of tachycardia, typically evidenced by a delay, without altering the atrial sequence upon administration of a His‐refractory PVC (with QRS fusion), or during the transition zone of VOP, suggests the existence of an AP with ventricular insertion. These observations rule out JT, AT, NF‐ORT, and AVNRT with or without a bystander atrioventricular AP. Nevertheless, ORT via any concealed AP with ventricular insertion and AVNRT with a bystander NVP remain candidates. It is important to note that the reset or termination of tachycardia without atrial capture 1 cycle after the His‐refractory PVC is specifically diagnostic of AVNRT with a bystander NVP (Figure 12B).^42^, ^45^ Theoretically, it is implausible that His‐refractory PVC resets or terminates the ORT without affecting the following His electrogram.

Step 2 involves the exclusion of ORT, demonstrating that the His bundle, AP, or both are outside the tachycardia circuit. AH block during the tachycardia excludes ORT. Additionally, the disappearance of the tachycardia reset phenomenon upon the delivery of a PVC earlier than the PVC satisfying Step 1, or an early PVC that captures the His electrogram antidromically (paradoxical reset phenomenon), also eliminates ORT. This phenomenon suggests either the sustainment of tachycardia despite AP block or dissociation of the His bundle from the tachycardia. However, this phenomenon has also been observed in a few NV‐ORT cases. This occurrence can arise because the reset may be concealed if the prematurity of the PVC matches the delay in ORT caused by the PVC. Therefore, although this phenomenon suggests the presence of AVNRT with a bystander‐concealed NVP, it may not be specific for diagnosis.

Step 3 involves confirming the AVN as the insertion site for the NVP according to the specific form of AVNRT and the sequence of reset/termination (Figure 12A). In fast‐slow AVNRTs with a bystander NVP commonly attaching to the SP, the sequence of atrial reset/block precedes the His reset/block upon delivery of the His‐refractory PVC (Figure 12B). In contrast, in slow‐fast AVNRTs with the NVP attaching to the SP, the His reset/block precedes the atrial reset/block. These observations stem from the difference in conduction direction over the SP between the two forms of AVNRT. As the SP functions as the retrograde limb in fast‐slow AVNRT, the His‐refractory PVC conducts retrograde to the SP through the NVP, leading to the resetting of the atrial electrogram before that of the His electrogram. In contrast, the SP acts as the anterograde limb in slow‐fast AVNRT, causing the His‐refractory PVC to conduct anterogradely to the SP and reset the His timing preceding the atrial timing. Similarly, in the context of slow‐fast AVNRTs with the NVP connecting to the FP, as the FP acts as the retrograde limb in slow‐fast AVNRT, the His‐refractory PVC conducts retrogradely to the FP through the concealed NVP, resulting in the atrial reset/block before the His reset/block (Figure 12C).

With these new insights, the diagnostic criteria for NVP‐related tachycardia have been fundamentally developed.

#### 1.3.5 ORT via a concealed HVP

In the final phase of the adventure in diagnosing narrow QRS tachycardia, Chung and Tchou et al. and Higuchi and Scheinman et al. documented ORTs via concealed HVP in their limited case series.^46^, ^47^, ^48^ Their reports emphasized the importance of continuous recording from the His bundle to the RB branch using a multipolar catheter. During ORT via a concealed HVP, an eccentric His and RB sequence (Chevron pattern) with a shorter HV interval compared to sinus rhythm was noted, marking the recognition of a concealed HVP as the first step in diagnosing HV‐ORT. From this perspective, distinguishing HV‐ORT from fascicular ventricular tachycardia and bundle branch reentrant tachycardia is crucial. They further highlighted the VOP from the RV basal septum alongside the RV apex, proposing that the PPI − TCL might differentiate HV‐ORT from these fascicular‐related tachycardias, with an estimated cutoff value of <50 ms, a value that could be adjusted by future large‐scale studies (Figure 12D).

## 2 CONCLUSIONS

As diagnostic precision increases, new challenges may arise, unseen in the era of lower resolution. Although each of these maneuvers is discussed separately, it is important to understand that multiple maneuvers are often required to make a correct diagnosis and there are some cases where a clear diagnosis is never achieved. The adventure of electrophysiologists is endless.

## CONFLICT OF INTEREST STATEMENT

Authors declare no conflict of interests for this article.

## APPROVAL OF THE RESEARCH PROTOCOL

The review board of Nihon University Itabashi Hospital and the review board of each participating center approved the data collection and analysis.

## PERMISSION TO REPRODUCE MATERIAL FROM OTHER SOURCES

- Pacing Clin Electrophysiol. 2022;45:839‐852.
- JACC Clin Electrophysiol. 2020;6:1797‐1807.
- JACC Clin Electrophysiol. 2024. doi: https://doi.org/10.1016/j.jacep.2024.02.030
- J Cardiovasc Electrophysiol. 2019;30:2528‐2530.
